# Maternal filicide in a cohort of English Serious Case Reviews

**DOI:** 10.1007/s00737-018-0820-7

**Published:** 2018-03-02

**Authors:** Peter Sidebotham, Ameeta Retzer

**Affiliations:** 1 Warwick Medical School, University of Warwick, Coventry, CV4 7AL UK; 2 Centre for Patient Reported Outcomes Research, Institute for Applied Health Research, University of Birmingham, Birmingham, B15 2TT UK

**Keywords:** Filicide, Fatal child abuse, Domestic violence

## Abstract

A national mixed-methods study of English Serious Case Reviews (SCRs) was carried out to better understand the characteristics and circumstances of maternally perpetrated filicides, to compare these with paternally perpetrated cases, and to identify learning points for mental health professionals. Published reports for all SCRs of children in England dying as a result of abuse or neglect from 2011 to 2014 were subject to qualitative analysis using a system of layered reading and inductive thematic analysis, along with descriptive and comparative quantitative analysis. There were 86 deaths directly attributable to child maltreatment within the immediate family. The mother was the suspected perpetrator in 20. Twelve of the mother perpetrators were victims of domestic violence, while 15 of the father perpetrators were known to be perpetrators of domestic violence. Those deaths resulting from impulsive violence or severe, persistent cruelty are almost exclusively perpetrated by males, while those with an apparent intent to kill the child are slightly more likely to be perpetrated by mothers. Four key themes were identified through the qualitative analysis: domestic violence, maternal mental illness, separation and maternal isolation, and the invisibility of the child. These findings highlight the important role of domestic violence and its interaction with maternal mental health. Professionals working with mothers with mental health problems need to adopt a supportive but professionally curious stance, to be alert to signs of escalating stress or worsening mental ill-health, and to provide supportive and accessible structures for at-risk families.

## Introduction

Filicide—the murder of a child by their parent—is a rare but tragic event that occurs across geographic and social boundaries. Differences in definitions and categorisation, along with the hidden nature of many cases, make incidence estimation difficult (Putkonen et al. 2016). Different approaches to classification have stemmed from psychology, drawing on the characteristics and motivations of the perpetrators, and from sociology, child protection, and paediatrics, drawing more on the victims’ characteristics and the circumstances of the death (Bourget et al. 2007; Brown and Tyson 2014; Putkonen et al. 2016; Resnick 1969; Sidebotham 2013). Both mothers and fathers are known to kill their children, with individual studies finding different relative proportions and characteristics (Bourget et al. 2007).

Various perpetrator characteristics have been identified, including parental mental illness, psycho-social stressors, social isolation, domestic violence, and parental adverse childhood experiences (Bourget et al. 2007). Understanding these characteristics within different parent groups is crucial to any prevention of filicide. As Putkonen et al. (2016) state, different sub-groups of potential perpetrators require different preventive approaches: strategies that work for violent, impulsive parents are unlikely to be effective among socially isolated and depressed but sober, or psychotic parents, for example.

To better understand the characteristics and circumstances of maternally perpetrated filicides, we analysed data from English Serious Case Reviews (SCRs) from 2011 to 2014. In England, there is a statutory requirement for Local Safeguarding Children Boards (LSCBs) to undertake a SCR whenever a child (from birth to their 18th birthday) dies and abuse or neglect is known or suspected (irrespective of whether the abuse or neglect was the primary cause of death, or whether the abuse or neglect was known prior to the child’s death) (HM Government 2015). These SCRs are independent multi-agency case reviews focused on learning lessons about how organisations are working together to safeguard and promote the welfare of children. There is a requirement that these reviews should be published, unless there are good reasons not to, such as protecting the welfare of surviving children, or because of ongoing court processes. Published reviews are available on individual LSCB websites and on a national repository held by the National Society for the Prevention of Cruelty to Children (NSPCC). The primary aim of this study, which was embedded within a wider study of all SCRs, was to analyse the circumstances and background features of cases of maternal filicide to identify factors that may help prevent future child deaths. Secondary aims were to compare the circumstances and background features of cases of maternal filicide with those of paternal filicide, and to identify learning points for mental health professionals.

## Materials and methods

Data were obtained for all SCRs notified to the Department for Education (DfE) relating to child deaths in England between 1 April 2011 and 31 March 2014. Basic demographic data and initial details of the circumstances of the death were provided by DfE. This included the ages and gender of the children, their ethnicity, and the Local Authority area in which they were resident. These cases were matched to published SCRs on the NSPCC national case review repository (NSPCC 2017), individual LSCB websites, or reports provided by LSCBs.

Each SCR was subject to a process of ‘layered reading’ (Brandon et al. 1999) to supplement the quantitative data and to develop a preliminary coding frame for qualitative analysis. Each case was categorised using our previously developed classification for violent and maltreatment-related deaths (Table 1) (Sidebotham et al. 2016). Cases where deaths were directly attributable to child maltreatment within the immediate family were included. Cases were excluded where the suspected perpetrator was outside the immediate family and where child maltreatment was not the primary cause of death. The relationship of the suspected perpetrator to the child was identified, where possible, from the information in the SCR report. Where one or more person(s) had been convicted of the child’s murder/manslaughter, this was taken as the suspected perpetrator; otherwise, this was based on the most likely perpetrator from the case information provided, or left as ‘unclear’ if there was insufficient information. For the purposes of this study, the immediate family was taken to be the birth or adoptive mother or father with whom the child was living, plus any partner of the birth or adoptive mother or father, and any siblings; parents were taken to be the biological parents of the child; and non-biological parent figures and partners of a biological parent are referred to as such.

**Table 1:** Classification system used for categorising violent and maltreatment-related deaths (Sidebotham et al. 2011; Sidebotham et al. 2016)

Category^a^	Criteria for inclusion
Overt filicide	Deaths where a child is killed by a parent or parent figure using overtly violent means, or with no attempt to conceal the fact of homicide, and where there appears to have been some intent to kill or harm the child. This includes multiple or extended familicide, or where the suspected perpetrator takes or attempts to take his/her own life. Includes deaths in fires with suspicion of arson and the suspected perpetrator is a parent/parent figure. Includes deaths from stabbings and firearms, or severe assaults with evidence of intent to kill the child.
Covert filicide	Deaths where a child is killed by a parent or parent figure but using less overtly violent means, and with some apparent attempt to conceal the fact of homicide, and where there appears to have been some intent to kill or harm the child. Includes deaths from abandonment, poisoning, drowning, suffocation, or asphyxiation. Includes deaths of newborn babies following concealed pregnancies and deliveries
Fatal physical abuse	Deaths following severe physical assaults (non-accidental injuries) where the suspected perpetrator is a parent or parent figure, and where there is no clear intent to kill or harm the child. Includes deaths from non-accidental head injuries (shaking or shaking-impact injuries), abdominal injuries, and multiple injuries. May include deaths where an implement has been used, but without evidence of intent to kill or harm the child.
Severe, persistent child cruelty	Deaths where a child dies as a result of a physical assault or neglect, and in which there is evidence of previous severe and persistent child cruelty. Includes deaths where a post-mortem examination reveals evidence of previous inflicted injuries (e.g. healing fractures) or long-standing neglect in addition to the primary cause of death, and children who have previously been on a child protection plan because of identified physical or emotional abuse or neglect.
Extreme neglect/deprivational abuse	Deaths where the child dies as a result of severe deprivation of his/her needs with evidence that this has been deliberate, persistent, or extreme. Includes deaths as a result of heat or cold exposure, starvation, or extreme, deliberate withholding of basic health care. Exclude deaths in which the neglect appears to be a reflection of parental incompetence, related to learning difficulties, physical or mental ill health, socio-economic deprivation and lack of access to services, or other environmental circumstances.
Child homicide (extra-familial)	Deaths where a child is killed by someone other than a parent or parent figure using overtly violent means, or with no attempt to conceal the fact of homicide, and where there appears to have been some intent to kill or harm the child. Includes deaths in fires with suspicion of arson and the suspected perpetrator is someone other than a parent/parent figure. Includes deaths from stabbings and firearms, or severe assaults with evidence of intent to kill or harm the child. Includes deaths following sexual assaults by a non-parent perpetrator. May include gang-related violence where there appears to have been intent to kill the specific victim, but excludes more general gang-related violence.
Fatal assault (extra-familial)	Deaths following severe physical assaults where the suspected perpetrator is someone other than a parent or parent figure, and where there is no clear intent to kill or harm the child. Includes peer-on-peer violence without evidence of intent to kill. Includes gang-related violence without evidence of intent to kill the victim
Deaths related to but not directly caused by maltreatment	There are a large number of deaths which are felt to be related to maltreatment, but in which the maltreatment cannot be considered a direct cause of death. Includes sudden unexpected deaths in infancy (SUDI) with clear concerns around parental care but where the death remains unexplained or is attributed to a natural cause. Includes fatal accidents where there may be issues of parental supervision and care, including accidental ingestion of drugs or other household substances, drownings, falls, electrocution, gunshot wounds, and fires. Includes those children dying of natural causes whose parents may not have sought medical intervention early enough. Includes deaths of older children with previous maltreatment, but where the maltreatment did not directly lead to the death, e.g. death from an overwhelming chest infection in a child severely disabled by a non-accidental head injury, suicide, or risk-taking behaviours including substance abuse in young people with a past history of abuse

^a^Each category is mutually exclusive; having reviewed the circumstances of the death, the research team applied the category that best fitted the circumstances

Cases of maternal overt or covert filicide were subject to a second and third round of layered reading using principles of inductive thematic analysis (Braun and Clarke 2006). The circumstances of the death and the familial and environmental background factors were manually coded. The information obtained was dependent on what was included in the published Serious Case Reviews, and we did not have access to any original case files. Child, parent, and family characteristics were included if they were mentioned in a SCR. As such, there were no specific criteria for any variables such as drug and alcohol misuse, parental mental ill health, or social isolation. The data were then reviewed to identify consistencies and discontinuities in the emerging themes.

Quantitative data were analysed using IBM SPSS Statistics 24. Where appropriate, chi-square tests, Fisher exact tests, and Student *t* tests were used to compare the maternal with the paternal filicide cases.

The data obtained for this analysis were from published, anonymised reports available in the public domain. Ethical approval was obtained from the University of East Anglia Research Ethics Committee. This project adhered to the University of East Anglia’s and the University of Warwick’s Research Codes of Conduct and to the Department for Education and the Department of Health Research Governance Framework for Health and Social Care.

## Results

**Fig. 1:**
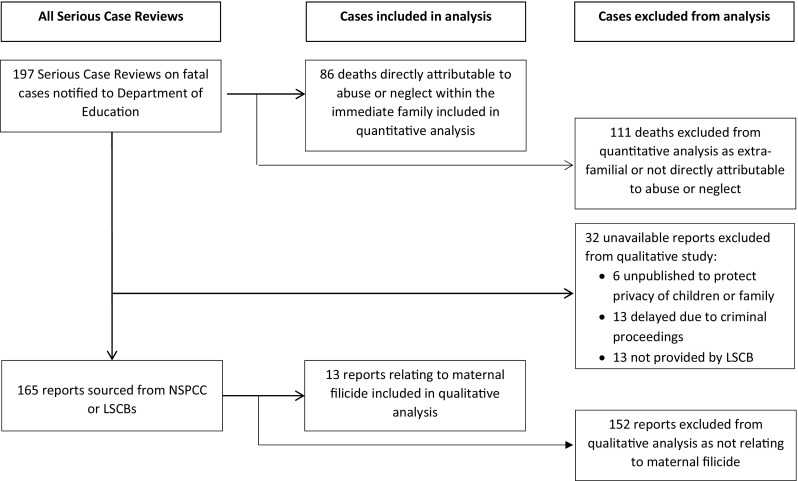
Serious case review data available for analysis

The DfE was notified of 197 SCRs for children who had died between 1 April 2011 and 31 March 2014. A published report was available for 165 (83.8%). Six SCRs had not been published to protect the privacy of surviving children or other relatives; for 13, publication had been delayed, primarily because of ongoing criminal proceedings; for the remaining 13, no report had been published and no response was obtained from the LSCB (Fig. 1).

Eighty-six cases (43.7%) were directly attributable to abuse or neglect within the context of the immediate family; 7 (3.6%) were attributed to extrafamilial homicide or fatal assaults; 91 (46.2%) were related to but not directly caused by child maltreatment; and 13 had insufficient information to categorise the death, or appeared unrelated to child maltreatment (Table 2).

**Table 2:** Category of death and suspected perpetrator as identified in the SCRs (whole cohort)

Category of death	Suspected perpetrator						
	Mother	Father	Father figure/mother’s partner	Both parents	Other family members (siblings, grandparents, aunts, or uncles)	Not clear	Total
Intrafamilial, direct maltreatment deaths							
Overt filicide	12	10	1	1	0	0	24
Covert filicide	3	0	0	0	0	0	3
Fatal physical abuse	2	11	9	2	3	12	39
Severe, persistent child cruelty	0	4	4	3	0	4	15
Extreme neglect/deprivational abuse	3	0	0	2	0	0	5
Subtotal	20	25	14	8	3	16	86
Child homicide/fatal assault (extrafamilial)	0	0	0	0	7	0	7
Death related to maltreatment	18	6	0	17	39	11	91
Not maltreatment related/category not clear	1	1	0	0	4	7	13
Total	39	32	14	25	53	34	197

The mother was the suspected perpetrator in 20 (23.3%) of the 86 deaths directly attributed to child maltreatment within the family; the father in 25 (29.1%); an unrelated male in 14 (16.3%); and both parents in eight (9.3%). In three cases, the suspected perpetrator was another close relative, and in 16 cases the suspected perpetrator was unclear.

Table 3 compares the characteristics of direct maltreatment deaths where the suspected perpetrator was the mother (*n* = 20) with those where it was the biological father (*n* = 25). Of the 12 overt filicide cases perpetrated by mothers, four involved the mother killing all children in the family and in a further six the victim was an only child. In all 12 cases, the mother committed or attempted suicide.

**Table 3:** Case characteristics identified from the SCRs for maternal and paternal filicide and direct fatal maltreatment cases

Characteristic	Maternal filicide	Paternal filicide	Statistical significance
Incident characteristics			
Familicide (multiple family members killed)	4/20 (20%)	6/25 (24%)	Not significant
Perpetrator suicide/attempted suicide	12/20 (60%)	8/25 (32%)	Not significant
Child characteristics			
Age in months: mean (SD) Median (range)	46.8 (50.3) 32 (0–202)	38.3 (43.0) 19 (1–137)	Not significant
Gender of child female	11 (55%)	14 (56%)	Not significant
Non-white ethnicity	9/19 (47.4%)	4/25 (16%)	Chi-square = 5.10, *p* = 0.024
Child known to children’s social care	14/18 (78%)	11/23 (48%)	Not significant
Parent characteristics			
Parental alcohol or drug misuse	5/20 (25%)	9/25 (36%)	Not significant
Parental mental health problems	14/18 (78%)	11/21 (52%)	Not significant
Known domestic violence	12/18 (67%)	15/21 (71%)	Not significant
History of violent crime	0/18 (0%)	6/21 (29%)	Fisher exact test, *p* = 0.022
Parental separation	13/18 (72%)	6/21 (29%)	Chi-square = 7.39, *p* = 0.007
Social isolation	4/18 (22%)	1/21 (5%)	Not significant

Not all information was available for all cases; the denominator for each factor reflects those cases for which information was available

Children killed by their mothers were more likely to be of Black and Minority Ethnic origin; their parents were more likely to be separated; and father perpetrators were more likely to have a history of violent crime. Over two thirds of cases occurred in a family context of known domestic violence. However, the cases varied in that 12 of the mother perpetrators were themselves victims of domestic violence, while 15 of the father perpetrators were known to be perpetrators of domestic violence. Other differences between cases perpetrated by mothers and fathers did not reach statistical significance.

### Qualitative analysis

Full SCR reports were available for 10 of the 12 maternally perpetrated overt filicides and for all three covert filicides. These reports were analysed thematically and four core themes identified: domestic violence, maternal mental illness, separation and maternal isolation, and the invisibility of the child. Sub-themes were identified for each of these themes, and in all there were exceptions that did not fit the theme (Table 4).

**Table 4:** Themes and subthemes from qualitative analysis of the SCRs

Theme	Subtheme	Examples^a^
The role of domestic violence	Emotional and sexual violence and coercive control as well as physical assaults	There was an alleged incident described as of ‘significant violence leaving visible marks, injuries and bruises to Mother’ with Father. [Case D] Mother alleged that her husband had been aggressive and thrown objects in anger, though never directed toward anyone. She was clear that he had never physically attacked her or the children and said she recognised the children’s need for a father. [Case E] Child F’s mother alleged physical violence and sexual harassment from her husband and in laws and stated that the laceration on her thigh had been inflicted with scissors by her husband. She alleged that her father-in-law had sexually molested her and had threatened to rape Child F and she said that she did not want to go back home as she was worried about the safety of her daughter and herself. [Case F]
	Impact of domestic violence on mother’s mental health and stress	A review of the entire multi-agency chronology indicated that the deterioration in the mother’s mental health was potentially linked with an increase in the occurrence of domestic abuse incidents. [Case G] Consideration was also given to mother’s mental health in light of the self harm. It was felt that this had been a reaction to her domestic situation and as the relationship was over, there were no ongoing concerns. The meeting felt that the risks to the mother by her ex partner had been addressed by the support provided, mother’s cooperation, her focus on the children and the bail conditions. [Case C]
	Mixed picture of allegations and counter allegations	Father offered an alternative account (later backed up by his elder daughter) indicating that mother had attacked him on the occasion in December 2012. [Case E] Child F’s father denied the allegations of domestic violence and alleged that Child F’s mother had Post Natal Depression and was aggressive, possessive, controlling, and drank whisky prior to their marriage. [Case F] Father categorically denied any domestic violence and pointed to the fact that he has no previous record of any kind. Father expressed strong feelings about the fact that he had been scrutinised when it was Mother, who needed help. [Case D] [Father] gave background information about the parents’ relationship… stating that the marriage had broken down due to the mother’s controlling and bullying behaviour and her dishonesty. His account was that he had separated from her during the pregnancy… but remained supportive. [Case H]
The role of maternal mental health and illness	Extremes of mental ill health were rarely known about before the fatal event	At a very early stage of intervention, there was a mental health assessment… which identified no presentation of depression or anxiety in the mother, and she denied ever experiencing these. Overall there was no presentation of concerns about mental illness and therefore it was concluded there was no role for mental health services. [Case I] In summary, there were no reported or presenting concerns around Child J’s emotional, social or behavioural development and contact with agencies were not deemed to be out of the ordinary or beyond universal service provision. [Case J] (covert)
	Family members may have had concerns about the mother’s mental well-being	The family noted that mother’s behaviour became increasingly watchful of Child B, and that she would ‘inspect her’ if she had been out of her sight. [Case B] Maternal Grandmother gave examples of Mother‘s distressed behaviour in the weeks over the summer, when she had been staying with them for several weeks and during the autumn. The maternal family related Mother ‘s behaviour as similar to post traumatic stress behaviour, sudden panic attacks, curling up in a foetal position and going out and then panicking and not being able to manage Baby D and calling up for help. [Case D]
	Delusional thoughts, extreme anxiety, and paranoia may be pointers of imminent risk	The Mental Health workers concluded that there was evidence of symptoms of mental disorder with overvalued ideation, delusions of reference and hypersensitivity to environmental dangers. However, mother was not responding to hallucinations, thought blocking or formal thought disorder. Mother declined any input from mental health services and it was agreed that as mother was not appropriate for services, the referral would be closed to the hospital trust. [Case B] During the preceding months, Child K had a history of severe anxiety disorder with some panic attacks and some limited depressive symptoms. She had been receiving treatment from her GP as well as various other health professionals and agencies. [Case K]
The role of separation and maternal isolation	Many, but not all, of the mothers were relatively isolated from their family and community	Records suggest that mother was fairly isolated from her family, and that she experienced some disharmony with her neighbours… Mother was a single mother, isolated from her family and her partner was abroad. [Case B] When Child F was two months old her mother made allegations to the Police against her husband and in laws and she left the family home, taking Child F with her. Child F and her mother moved into a house provided by a women’s refuge in Bradford and remained in Bradford for the remainder of Child F’s life… It is believed that she had never been to Bradford before and did not know anyone in the city or the surrounding area. As a non UK citizen who was resident in the UK due to her marriage she did not have an immediate entitlement to remain in the UK in her own right and had no recourse to public funds and therefore no independent source of income. [Case F]
	Parental separation may be a trigger for increased maternal isolation and stress	Child L lived with her mother and, for most of her life, her father was also resident in the family home. There were periods where her parents separated and it has been confirmed that the latest separation took place approximately two weeks before Child L's death. On that day, she and her mother were living alone. [Case L]
The invisibility of the child	The children did not stand out as being particularly vulnerable or at risk	Baby D was a seven month old baby, who was described in records as well cared for, healthy and reaching all developmental milestones. [Case D] Child L herself was a bright and attractive child with a charming smile. In all senses her development was at least age-appropriate, and no-one who knew the family ever had the slight concern for her safety or welfare prior to the incident that led to her death. [Case L]
	The relationship between the mother and the child was described as loving and warm	Through speaking with the family, a picture emerges of Child B as a cherished child, physically well cared for, whose primary attachment figure was mother. [Case B] Baby D and the interactions between Baby D and Mother were consistently described in a positive way with descriptions of warm and affectionate interactions and appropriate responses by Mother to Baby D. [Case D]
	Love for the child as a motivation for killing the child	We can now never be certain what were the key factors in her decision to take Child L’s life, but there is an important clue in one of her suicide notes where she writes ‘I can’t leave her here, but I can’t stay’. It appears that Child L was not killed because she was unwanted, but because she was deeply loved and her mother could not envisage a life for her when she herself was dead. [Case L]

^a^In the example quotations, we have applied our own case initials to each case. All the data included here are direct quotes from the published SCRs. As such, all this information is already in the public domain

#### The role of domestic violence

In nine cases, including one case of covert filicide, the mother had made domestic violence allegations including direct physical assaults, and sustained emotional and sexual violence and coercive control. This culture of violence was noted to have a direct impact on mothers’ health and well-being, and in some cases was directly linked to deteriorating mental health or to self-harming behaviour. In one case, ongoing domestic violence-related court proceedings precluded the provision of mental health services for the mother. In other cases, it appeared that a focus on mothers’ immediate physical safety and her separation from the perpetrator meant her parenting capacity was overlooked. However, in six of the nine cases, partners denied the veracity of the allegations or made counter allegations of violence or controlling behaviour by the mother.

#### The role of maternal mental health and illness

While maternal mental illness was highly prevalent in these cases, severe mental illness was rarely identified before the fatal event, and in many cases, there were no indicators of significant mental illness. In some cases, viewed in retrospect, signs were missed by professionals, including indicators of delusional or paranoid thinking. In others, concerns had receded by the time the mother was assessed. With hindsight, relatives also identified areas of concern, though these had rarely been sufficient for them to seek help. The difficulties in identifying clear pointers of risk were raised by a psychiatric expert reviewing one case:

Her view is that it is more likely that [mother] had a more longstanding serious mental illness, possibly a chronic psychotic state which she was able to contain by her unusual lifestyle, which might have merely seemed eccentric to others, except at moments of great stress. [The doctor] has suggested that this would explain her fluctuating presentation and the fact that on occasion of contact with professionals she appeared to have none of the symptoms that a non-specialist would regard as indicative of a psychosis i.e. she did not appear to have hallucinations or thought disorder. Any evidence of delusional thinking or unreasonable beliefs would only have been evident if the reasons had been explored and then probably only by an experienced mental health practitioner. [Case A]

#### The role of separation and maternal isolation

Many mothers appeared isolated from their family and community. Among those who were isolated, this could be compounded by relationship breakdown, often precipitated by domestic violence. Separation from an abusive partner may result in safety for the mother and child but could also increase her sense of isolation and distress. One mother’s experience of emotional isolation due to her partner’s infidelity was considered a key factor in the mother’s stress and deteriorating mental health.

#### The invisibility of the child

With four important exceptions, the children killed by their mothers were previously unknown to social services as being at risk of harm. To professionals, they presented as healthy, thriving children with no indicators of concern. That their families were ‘well-educated’, ‘middle class’, and enjoyed ‘a good standard of living’ was specifically mentioned in some reviews. The relationship between the mother and the child was typically perceived—by professionals and other family members—as loving and warm, with the mother responding well to the physical and emotional needs of the child. In one case, it was postulated that the mother’s love for the child was a primary motivation for her taking the child’s life before committing suicide.

Of the three covert filicide cases, one involved a teenage mother with a concealed pregnancy who had suffocated her baby immediately after birth. There had been no previous concerns about the mother who was described as ‘an articulate and intelligent young woman’. She had not presented with any typical signs of pregnancy and denied that she was pregnant. The other two cases involved older women, one of whom had not presented with any prior concerns.

There were, however, four cases of maternal filicide where child welfare concerns had been raised before the fatal event. In one case, the child was subject to a child protection plan following a serious physical assault by the mother. In another, a child protection investigation had been initiated after the 5-year-old disclosed being slapped by his parents and witnessing arguments at home. In the third, long-standing concerns about the well-being of all the children in the family resulted in a series of child protection plans and interventions which were, however, limited by poor parental engagement. In the fourth, there were concerns of maternal ambivalence toward her child, and fears that she might abscond with the child to another country. A striking feature of all these cases was the mothers’ awareness of the involvement and impact of social services, and their fear of their child’s removal. This fear may have precipitated the fatal actions of one mother:

[Mother] went on to say that she ‘did what she did because it needed to be done’, and that ‘the system was corrupt; Social Workers were treating her badly and had taken her daughter’… [Mother] informed medical staff that she was dead at home because she had suffocated her. [Case B]

Another mother’s previous engagement with social services had resulted in her presenting a positive appearance thus avoiding arousing concern—a form of disguised compliance:

The contribution of family members and friends was extremely helpful, both in terms of understanding what the children and their mother were like, as well as understanding the mother’s experiences of what it was like to be involved with the services who were working to support her and the children. There were several key factors that came through in these conversations. The first and overriding message was that the children’s mother was a loving, caring and competent mother, who in her normal life would do anything to protect her children. The next was that she was very keen to display a positive aspect to anyone in a position of authority; she was very able to understand and display what was expected of her. Several of those we spoke with indicated that while she was able to display this positive aspect, because of early interactions with children’s services, she was in fear that if she did not do what was expected of her, her children might be taken back into the care of the local authority. [Case C]

## Discussion and conclusions

In this national cohort of child maltreatment fatalities, males were the most common perpetrators of deaths directly caused by maltreatment. Nevertheless, mothers were implicated, alone or with their partner, in nearly one third of cases. This is similar to our previously published national analysis (Sidebotham et al. 2011). In comparing mothers with fathers or father figures, some important findings emerge. Those deaths resulting from impulsive violence or severe, persistent cruelty are almost exclusively perpetrated by males, while those with an apparent intent to kill the child (overt or covert filicide) are slightly more likely to be perpetrated by mothers. In keeping with this, paternal perpetrators were more likely to have a history of violent crime, suggesting that violence and impulsivity are prevalent characteristics. In contrast, none of the mothers had such a history.

It was striking that in nearly all maternally perpetrated filicides, the victim was either an only child or all children in the family were killed, and the mother committed or attempted suicide. In addition, the majority of mothers had underlying mental illness, and two thirds were victims of domestic violence.

These findings shed additional light on the nature and characteristics of maternal filicide, building on previous work (Bourget et al. 2007; Friedman et al. 2005; Mugavin 2005; Oberman 2003; Putkonen et al. 2016). In particular, our findings highlight the important role of domestic violence and its interaction with maternal mental health, echoing characteristics identified by Putkonen et al. (2016). Domestic violence was a common, though not universal, finding. While this often involved acts of physical violence, the pervasive nature of coercive control was clear (Stark 2007). However, many cases involved counter allegations of control and abuse perpetrated by the mothers. This highlights the damaging nature of these relationships where elements of violence, control, and domination can impact on the mothers’ emotional health and well-being and on their parenting. Separation of a mother from a violent partner may bring some immediate safety, but can also lead to increased stress and isolation. Some mothers may view filicide, together with suicide, as the only way of escape both for themselves and their children.

The role of maternal mental illness in these cases presents challenges to professionals. While a majority of the mother perpetrators had identifiable mental health problems, for many, these were not obvious prior to the fatal event. Our qualitative review demonstrated how, in many cases, the severity of the mothers’ mental health problems were unrecognised or did not lead to timely mental health assessments or intervention. This has implications for all those working in mental health, primary care services, and child and maternal welfare. Services must be accessible; concerns, including those expressed by relatives, should be taken seriously; and health and social care practitioners must be sensitive to signs indicating escalating stress or severity of mental illness, particularly where there are delusional thoughts relating to children, suicidal ideation, or self-harm.

It is important to note that most children in this cohort did not present as being at risk of harm. Practitioners should therefore focus on the wider family dynamics and parental risk indicators, rather than solely looking for evidence of harm in the children. This is even more important given that some mothers are particularly attuned to the possibility of their child’s removal and that this may be a factor in some filicides. This resonates with the conclusion of Oberman (2003) that ‘maternal filicide is committed by mothers who cannot parent their child under the circumstances dictated by their particular position in place and time’ (p494) and that ‘only when we come face to face with the desperation of these mothers can we begin to devise effective manners of protecting both them and their children’ (p514).

This study, drawing on a comprehensive national cohort of child maltreatment deaths provides a unique perspective on the nature and characteristics of maternally perpetrated filicides. Nevertheless, there are important limitations. While we are confident, because of the legislation surrounding the requirement for SCRs, that all cases were notified, this has not been independently verified, and there is currently no way of confirming whether this is indeed the case. We recognise that, even as a comprehensive national cohort, the number of cases included remains small, and some caution should therefore be applied in extrapolating from these findings. For our analysis, we were dependent on the information gathered for the SCR and published in the final report. We did not have access to any primary case files or coronial or legal outcomes in the cases. We were thus reliant on the information that the SCR authors chose to include in their reports, which could be subject to conscious or unconscious bias. The fact that a particular characteristic was not mentioned in a review could have been because it was not present, it had not been identified, or that the SCR author did not consider it significant. It is possible, therefore, that some characteristics had a higher prevalence than that recorded. In addition, there were no standard criteria for defining variables such as mental illness, and there could be considerable variation between SCRs as to what was or was not included within any such definition. The retrospective nature of this study meant that so we cannot provide comparator data for non-filicidal mothers. Even as a national cohort extending over 3 years, the number of maternal filicides is small, limiting the statistical significance of some of the observed trends. However, the addition of qualitative data strengthens the relevance and validity of these findings and contributes to our understanding of these cases.

Maternal filicides are, fortunately, rare. We identified less than five cases per year in England. They are extremely distressing, and those working in child and family welfare must do all we can to support families at risk, and to minimise those risks. In a systematic review of maternal filicide, Friedman et al. (2005) highlighted the limited state of knowledge of the maternal characteristics that distinguish mothers at risk of killing their children. The heterogeneous and often hidden nature of these risks emphasises that it will not be possible to fully prevent all maternal filicides. Nevertheless, a deeper understanding of the characteristics of these cases may facilitate strategies that help to minimise risk. Most importantly, practitioners must be aware of the impact of domestic violence on mothers and children, and the need to adopt a supportive but professionally curious stance; to be alert to signs of escalating stress or worsening mental ill-health; and to provide supportive and accessible structures for at-risk families.
